# Discovery of seed germinating fungi (*Mycetinis scorodonius*) from *Gastrodia elata* Bl. *f. glauca* S. chow in Changbai Mountain and examination of their germination ability

**DOI:** 10.1038/s41598-024-63189-3

**Published:** 2024-05-28

**Authors:** Xinyu Yang, Yugang Gao, Zhaochun Li, Pu Zang, Yan Zhao, Qun Liu

**Affiliations:** 1 College of Chinese Materia Medica, Jilin Agricultural University, Changchun, 130118 China; 2 Institute of Botany, Jiangsu Province and Chinese Academy of Sciences (Nanjing Botanical Garden Mem. Sun Yat–Sen), Nanjing, 210014 China; 3 Laboratory of Medicinal Plant Cultivation and Breeding, State Administration of Traditional Chinese Medicine, Changchun, 130118 China; 4 JINGZHEN TIANMA Co., Ltd., Jingyu County, Baishan, 135200 Jilin China

**Keywords:** *Gastrodia elata* Bl. *f. glauca* S. chow in Changbai Mountain, Sexual reproduction, Germinating fungi, *Mycetinis scorodonius*, Seed germination of *Gastrodia elata* Bl, Molecular biology, Plant sciences

## Abstract

Multi-generational asexual reproduction of *Gastrodia elata* Bl. will cause seedling species degeneration. Sexual reproduction of *Gastrodia elata* Bl. seed is an effective method to solve the problem of degeneration. The development of *Gastrodia elata* Bl. seeds cannot be separated from the germination fungus. However, there are few strains of germination fungus in production, and there is also the problem of species degradation in application for many years. It is very important for the sexual reproduction of *Gastrodia elata* Bl. to isolate more new strains of excellent germination fungus from the origin. This study used the *Gastrodia elata* Bl. *f. glauca* S. chow seeds germination vegetative propagation corms capture method to isolate its symbiotic germination fungus, and comprehensively identified the species of germination fungus by colony morphology, ITS, sporocarps regeneration and germination function, and compared the growth characteristics and germination ability with other germination fungus (*Mycena purpureofusca*, *Mycena dendrobii* and *Mycena osmundicola*). The germination fungus was isolated from the vegetative propagation corms of *Gastrodia elata* Bl. *f. glauca* S. chow seeds and named GYGL-1. After comprehensive identification, GYGL-1 was *Mycetinis scorodonius*. Compared with other germination fungus, GYGL-1 has fast germination speed, vigorous growth, and high germination ability for *Gastrodia elata* Bl. *f. glauca* S. chow seeds. Innovated the isolation method of *Gastrodia elata* Bl. seeds germination fungus, obtained the regenerated sporocarps of the germination fungus, and discovered that *Mycetinis scorodonius* has a new function of germinating *Gastrodia elata* Bl. *f. glauca* S. chow seeds, enriching the resource library of *Gastrodia elata* Bl. germination fungus.

## Introduction

*Gastrodia elata* Bl. is a bulk medicinal material, which is well–known in Jilin Province, China. It has traditional effects such as relieving wind and convulsions, calming the liver, dispelling wind, and dredging collaterals^1^. *G elata* Bl. also has modern pharmacological effects, such as antidepressant^2,3^, anticonvulsant^4,5^, anti–aging^6,7^, and neuroprotective effects^8,9^, with a thousand years of medicinal use. In 2019, the Chinese government announced the categorization of *G elata* Bl. as a “food and drug substance,” and the demand is increasing^10^. However, there is a serious shortage of wild *G elata* Bl. resources^11^. An effective way to solve the contradiction between supply and demand of *G elata* Bl. is artificial cultivation, and reproduction is the key to artificial cultivation.

**Figure 1:**
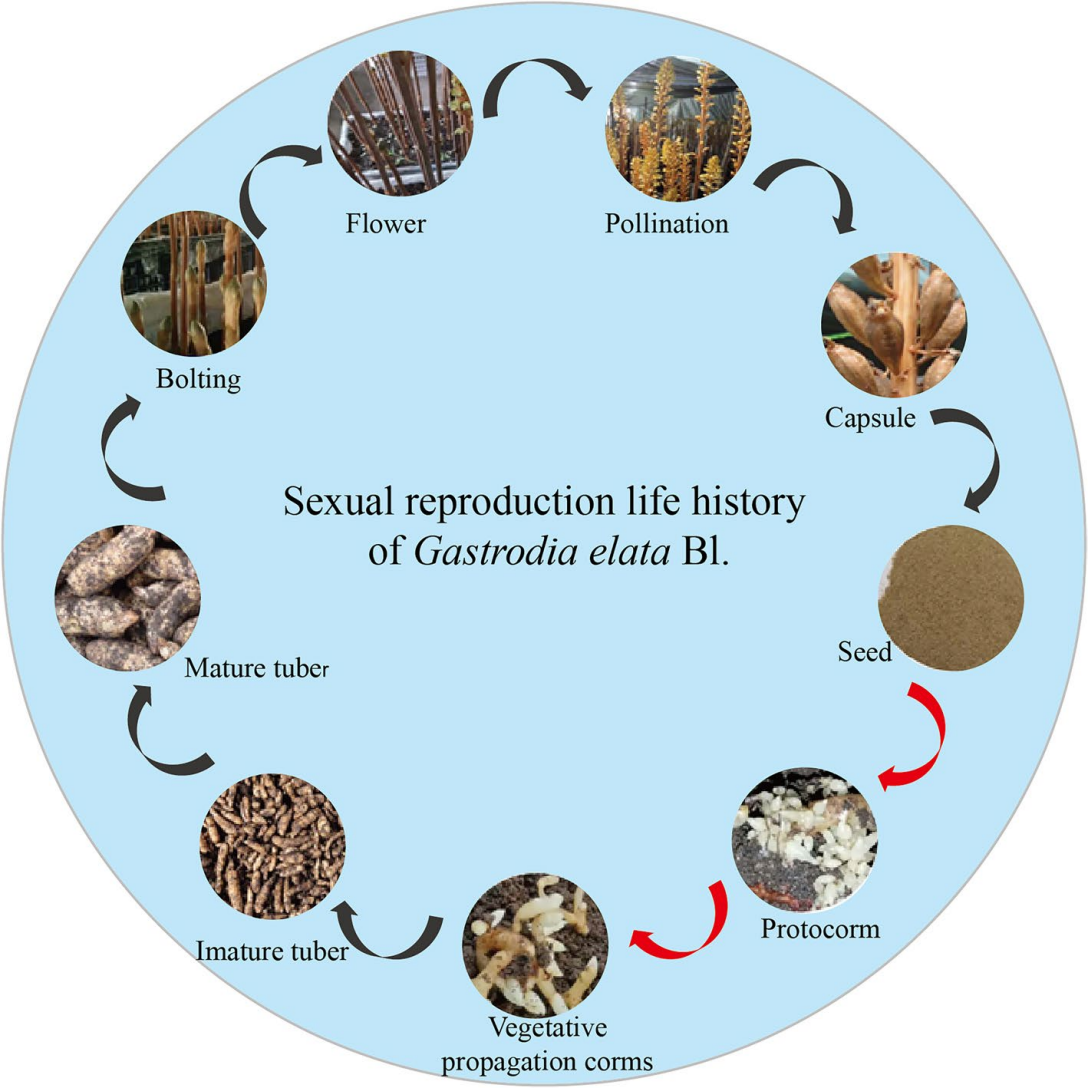
Sexual reproduction life history of *G elata* Bl. (The red arrow marks the stage that requires germination fungus).

Methods for reproduction of *G. elata* Bl. includes asexual and sexual reproduction. Asexual reproduction takes a short time to produce immature tuber seedlings, but multiple generations of asexual reproduction will cause degradation of immature tuber seedlings, resulting in thin, deformed and yellowish-brown *G. elata* Bl., slow growth, poor hyphal ability, easy to rot, yield reduction or even no harvest, etc.^12,13^. Compared to asexual reproduction, sexually reproducted immature tuber seedlings have strong resistance to adversity and vigorous vitality, and its cultivation of high quality and high yield of *G*. *elata *Bl., is currently an effective means of solving the degradation of *G. elata* Bl.^14^. As shown in Fig. 1, sexual reproduction involves at least nine steps: bolting, flowers, pollination, capsules, seeds, protocorms, vegetative propagation corms, immature tubers, mature tubers, and other growth and development stages^15^. In the process of sexual reproduction, the germination ability of seed is the most critical, but *G. elata* Bl. seeds are tiny, without endosperm and other nutrient storage organs, and must rely on nutrients supplied by germination fungus (*Mycena osmundicola*, *Mycena dendrobii*) to provide nutrients after infesting the embryo cells in order to germinate^16,17^.

*Gastrodia elata* Bl.* f*. *glauca* S. chow originates from northeast China and is one of the six varieties of *G. elata* Bl. It is a perennial heterotrophic herbaceous plant of the Orchidaceae family and is also an obligate fungal heterotrophic plant^18^. *G. elata* Bl. has different living habits in different producing areas, and the suitable germination fungus for the germination of *G. elata* Bl. seeds in different producing areas are also different. Therefore, it has become inevitable to isolate excellent strains suitable for the germination of local *G. elata* Bl. However, because the germination fungus are weak saprotrophs, their slow growth and difficult culture make isolation and identification more difficult^19^. Few strains were isolated and identified, mainly including six types of *Mycena osmundicola*, *Mycena dendrobii*, *Mycena anoectochila*, *Mycena purpureofusca*, *Mycena abramsii* and *Mycena citrinomarginata*^20–25^. After being used for many generations, the viability of fungi such as germination fungus were significantly reduced, resulting in a reduction in the germination rate of *G. elata* Bl. seeds or even no germination at all^26^. Obviously, it has become inevitable to isolate more and better germination fungus resources. In particular, the isolation of strains of origin suitable for germination of the *G. elata* Bl. *f. glauca* seeds is more important to ensure the yield and quality of *G. elata* Bl.

Therefore, the following studies were carried out: firstly, based on the characteristic that the germination of *G. elata* Bl. *f. glauca* seeds to grow into vegetative propagation corms depends on the symbiosis of germination fungus, the germination fungus were captured using the vegetative propagation corms germinated from *G. elata* Bl. *f. glauca* seeds sown in the wild as carriers. Secondly, the germination fungus was isolated and purified from the captured vegetative propagation corms using dilution separation method. Thirdly, the species of the germination fungus were identified by colony morphology, ITS and substrate regeneration. Finally, the growth rate of the isolated germination fungus and the germination ability of *G. elata* Bl. *f. glauca* seeds were evaluated. This study innovated the efficient targeting isolation method of germination fungus, enriched the resources of germination fungus of *G. elata* Bl., and has important theoretical significance and practical value for the sexual reproduction of *G. elata* Bl.

## Materials and methods

### Experimental materials

In April, the overwintering–treated mature tuber of *G. elata* Bl. *f. glauca* was cultivated at room temperature (20 °C). In May, after flowering, artificial pollination was performed to obtain *G. elata* Bl. *f. glauca* seeds. Selected seeds whose viability was greater than 95% measured by TTC method for experiments (Fig. S1). *M. purpureofusca*, *M. dendrobii*, *M. osmundicola* and *Armillaria gallica* were all identified and preserved by our laboratory. Experimental research and field studies on plants (either cultivated or wild), including the collection of plant material, in compliance with relevant institutional, national, and international guidelines and legislation.

### Methods for capturing germination fungus from the vegetative propagation corms of *G. elata* Bl. *f. glauca* seeds

**Figure 2:**
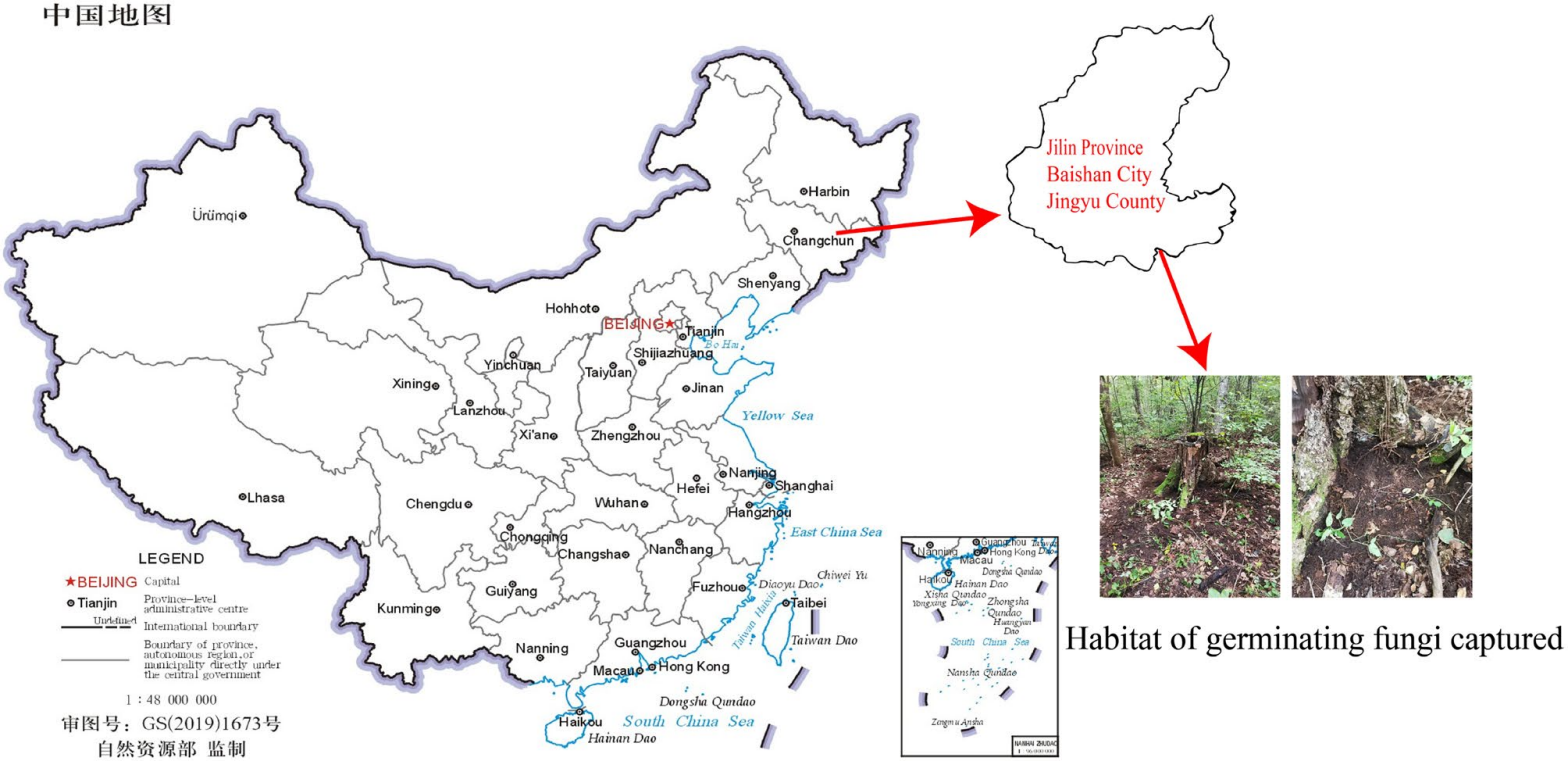
Habitat of germination fungus was captured (the map of China was compiled by China Map Publishing House and National Basic Geographic Information Center. The link is http://bzdt.ch.mnr.gov.cn/download.html?superclassName=%25E4%25B8%2596%25E7%2595%258C%25E5%259C%25B0%25E5%259B%25BE. The map of Jingyu County, Baishan City, Jilin Province was downloaded from the "Geospatial Data Cloud", the link is https://www.gscloud.cn/search).

After the seeds selected in 2.1 are sown under the birch trees in the woodland (Fig. 2), the naturally occurring germination fungus combined with the *G. elata* Bl*. f. glauca* seeds to germinate protocorms and continued to grow into vegetative propagation corms. Two months later, we searched for vegetative propagation corms at the sowing site and captured germination fungus symbiotic with the *G. elata* Bl*. f. glauca* seeds. The capture of germination fungus was carried out in Jingyu County, Baishan City, Jilin Province, China, which is located between longitude 126°30ʹ–127°16ʹE and latitude 42°06ʹ–42°48ʹN, close to the Changbai Mountain and Songhua River. The climate was a typical continental temperate monsoon climate, with an annual average temperature of 3.6 °C, an annual average precipitation of 700 mm, and a long and cold winter (according to the average temperature, winter was from November to April of the following year)^18^.

### Isolation and morphological characteristics identification of germination fungus from *G. elata* Bl. *f. glauca* seeds

Took the vegetative propagation corms of *G. elata* Bl. *f.* glauca collected in 2.2, washed them three times with sterile water, and blotted them dry with sterile filter paper. Soaked the vegetative propagation corms in 75% ethanol for 1 min and then washed them three times with sterile water. Cut the collected vegetative propagation corms into 1 × 1 mm small pieces, put a small piece of vegetative propagation corm together into a 1.5 ml centrifuge tube filled with sterile water, shook at 120 rpm for 10 min, mixed well, and diluted it into 10^3^, 10^4^, and 10^5^ times of the extraction solution respectively. Used an inoculating loop to dip the extracts of different dilution ratios inoculate into PDA slant medium and cultured them at 25 °C. Colonies will grow out in about 1–3 days. Selected a single colony with early germination and fast growth that has the morphology of germination fungus, transferred the colony to a new medium, and repeated this process three times. The purified germination fungus (named GYGLs) was used for observation and identification of morphology, color and growth rate. GYGLs was inoculated into PDA slant medium and stored at 4 °C (for short-term storage) and at − 80 °C with 20% glycerol (for long-term storage).

### Molecular identification of isolated germination fungus from *G. elata* Bl. *f. glauca* seeds

Took the purified germination fungus GYGLs, used the Fungal DNA Extraction Kit (Solarbio, Beijing, China) to extract strain DNA, and stored the extracted DNA at − 20 °C for the following experiment. PCR amplification and analysis of the ITS region of GYGLs DNA were conducted using fungal universal primers ITS1 (5ʹ‑TCC GTA GGT GAA CCT GCG G‑3ʹ) and ITS4 (5ʹ‑TCC TCC GCT TAT TGA TAT GC‑3ʹ). The PCR conditions were described as follows: a total of 25 μL volume including Taq PCR Master Mix, 12.5 μL; DNA template, 1 μL; ITS1, 1 μL; ITS4, 1 μL; ddH_2_O, 9.5 μL. The PCR conditions included initial denaturation (94 °C, 4 min), followed by 30 cycles of denaturation (94 °C, 30 s), annealing (55 °C, 30 s), extension (72 °C, 50 s), and the final extension was performed at 72 °C for 10 min. The amplified PCR product was detected by electrophoresis on a 1% agarose gel, and samples with a single, clear, bright band were subjected to sequencing (Sangon, Shanghai, China). To determine the biological classification status, the sequencing results were compared with NCBI data to identify the fungus with the highest similarity. The top 50 sequences with the highest homology were selected for phylogenetic tree construction using MEGA 7.0 software and the Maximum Likelihood method with 1000 bootstrap repeats.

### Regenerated sporocarps identification of isolated germination fungus from *G. elata* Bl. *f. glauca* seeds

Inoculated the GYGL-1 identified in 2.4 and cultured on PDA medium into the original seed culture medium (40% wood chips (*Quercus mongolica* Fisch. ex Ledeb.), 40% leaf fragments (*Quercus mongolica*), 18% bran, 1% gypsum, and 1% sugar, autoclaved at 121 °C for 2 h and then cooled naturally), cultured the bottle until it was full at 25 °C (about 10–15 days). Then took about 1 g of GYGL-1 from the original seed culture medium and inoculated it into the cultivation seed bag medium (15% wood chips (*Quercus mongolica*), 39% leaves (*Quercus mongolica*), 20% cottonseed husk, 20% bran, 3% soybean meal, 1% sugar, 1% gypsum and 1% lime, autoclaved at 121 °C for 2 h and then cooled naturally). Specification of the cultivation seed bag: inner polypropylene bag specification was 16.2 cm × 31 cm × 0.04 cm, outer polyethylene bag specification was 16.5 cm × 34 cm × 0.04 cm. After inoculation, placed it in a culture room at a constant temperature of 22–25 °C with light shielding for about 25–30 days (Fig. S2). After the GYGL-1 mycelium grew all over the bag, stimulated it at low temperature (8–10 °C) for 14 h. Then cut a 10 cm small opening in the plastic film of the bag to expose the mycelium and cultured it at 25 °C for 15 days to observe the morphology of the sporocarps and determine the species of GYGL-1.

### Germination function identification of GYGL-1

First, put some sterilized soil (20 g, thickness 1–1.5 cm) into the culture bottle (6 cm × 9.5 cm × 3.5 cm), and the *G. elata* Bl. *f. glauca* seeds (1 mg) were stirred well with the germination fungus (10 g) cultivated in the previous step and spread on the soil, and the germination fungus mixed with *G. elata* Bl. *f. glauca* seeds were covered with a layer of soil (20 g), and the process was carried out under aseptic conditions, and repeated in 6 bottles. Cultivated at a constant temperature (25 °C) for 8 weeks, to observe whether the *G. elata* Bl. *f. glauca* seeds had germinated into vegetative propagation corms.

### Comparison of growth rate between the GYGL-1 and other germination fungus

In order to screen for excellent germination fungus, different strains were cultured comparatively. First, the *M. purpureofusca,* GYGL-1, *M. dendrobii* and *M. osmundicola*, which had been frozen at − 80 °C, were inoculated on PDA culture medium and cultured at a constant temperature of 25 °C. The time required for germination was calculated from the day of inoculation until the appearance of tiny mycelium. Each treatment was repeated 6 times.

Secondly, statistical analysis was performed on the growth rates of different germination fungus on PDA culture medium and fungal bag culture medium. On the PDA medium, starting from the day of germination of the germination fungus, a vernier caliper was used to to measure the spreading distance (s) at the edge of the colony at the same time every day (t), and the growth rate (v = s/t) was calculated. On the fungal bag culture medium, the time until the mycelium grew over the fungal bag was recorded starting from the day of inoculation of the germination fungus. Each treatment was repeated 6 times.

Finally, 1 ml of fungal liquid in different logarithmic growth phases of the above four germination fungus (the fungus ball was broken with a magnetic stirrer, and the OD value were adjusted to 0.6) was taken. Inoculated into 30 ml of PDB medium, incubated at 120 rpm and 25 °C in the dark for 15 days, and the fungus ball was weighed after 15 days. Each treatment was repeated 6 times.

### Effect of GYGL-1 and other germination fungus on the germination ability of *G. elata* Bl. *f. glauca* seeds

In order to evaluate the germination ability of four different strains on *G. elata* Bl. *f. glauca* seeds, the *M. purpureofusca,* GYGL-1, *M. dendrobii* and *M. osmundicola* were cultured with *G. elata* Bl. *f. glauca* seeds respectively. The culture method was the same as 2.6 and was repeated for twelve bottles. Six culture bottles were selected at the fifth week of culture, and five randomly selected fields of view under a dissecting microscope were used to record the number of protocorms that had germinated from *G. elata* Bl. *f. glauca* seeds and the number of seeds that did not germinate, and to calculate the *G. elata* Bl. *f. glauca* seed germination rate (the number of germinated seeds as a percentage of the total number of seeds). In the eighth week of cultivation, the number and weight of vegetative propagation corms germinated from the remaining six bottles of *G. elata* Bl. *f. glauca* seeds were recorded and measured to evaluate their germination ability.

### Effect of GYGL-1 and other germination fungus on the germination ability and seedling growth of *G. elata* Bl. *f. glauca* seeds sown in farmland

Took the *M. purpureofusca,* GYGL-1, *M. dendrobii*, *M. osmundicola* and *G. elata* Bl. *f. glauca* seed, mixed them at a ratio of 1 mg of seeds and 10 g of germination fungus, and then sowed them in farmland according to conventional production methods. Each treatment was designed with six replicates, each with an area of 45 m^2^ (Fig. S3). The amount of sowing mixed seeds and germination fungus was 1.5 kg/m^2^, and the amount of *A. gallica* was 2.5 kg/m^2^. On the fifth week after planting, 60 g of soil where *G. elata* Bl. *f. glauca* seeds had been sown was randomly collected from each replicate, and five fields of view were randomly selected under a dissecting microscope to calculate the germination rate of *G. elata* Bl. *f. glauca* seeds as described above. In the eighth week after planting, 60 g of soil sown with *G. elata* Bl. *f. glauca* seeds was collected in each repetition, and the vegetative propagation corms were collected and tested for number and weight to evaluate the germination abality of each strain on *G. elata* Bl. *f. glauca* seeds. At the 18th month after planting, the soil sown with *G. elata* Bl. *f. glauca* seeds was collected in 1 m^2^ of each replicate, and immature tuber seedlings were collected, and tested for number and weight of immature tuber seedlings to evaluate the effect of each strain on immature tuber seedlings.

Farmland sowing *G. elata* Bl. *f. glauca* seed specific steps: in the farmland, dug a length of 30 m, 1.5 m wide, 7–8 cm deep long pit, in the bottom of the pit laid a layer of cut through the mouth of the fish scale mouth of the *Quercus mongolica* (diameter 5–6 cm, length 15 cm), spacing 2–3 cm, filled the gaps with soil. Placed *A. gallica* block at both ends and at the mouth of the fish scale (medium formula: 50% wood chips (*Quercus mongolica*), 25% corn, 10% cottonseed husk, 8% bran, 5% soybean meal, 1% gypsum and 1% lime, autoclaved at 121 °C for 2 h and then cooled naturally). Covered the *A. gallica* blocks with a layer of soil 2 cm thick. Evenly placed the germination fungus mixed with *G. elata* Bl. *f. glauca* seeds on top. Covered the germination fungus with a layer of 2 cm thick soil, and evenly placed the *Quercus mongolica* (3–4 cm in diameter, 15 cm long) on top of the soil, and placed *A. gallica* blocks in the gaps, filled with a layer of soil with a thickness of 7 ~ 8 cm, and compacted the soil slightly. Covered it with 7–8 cm thick *Quercus mongolica* leaves and covered it with a shade net.

### Approval for experiments

Experimental and field research on plants (cultivated or wild) in the manuscript, including the collection of plant materials, in accordance with relevant institutional, national, and international standards and legislation.

## Results and analysis

### Results of the isolation of seed germination fungus from *G. elata* Bl. *f. glauca* seeds

**Figure 3:**
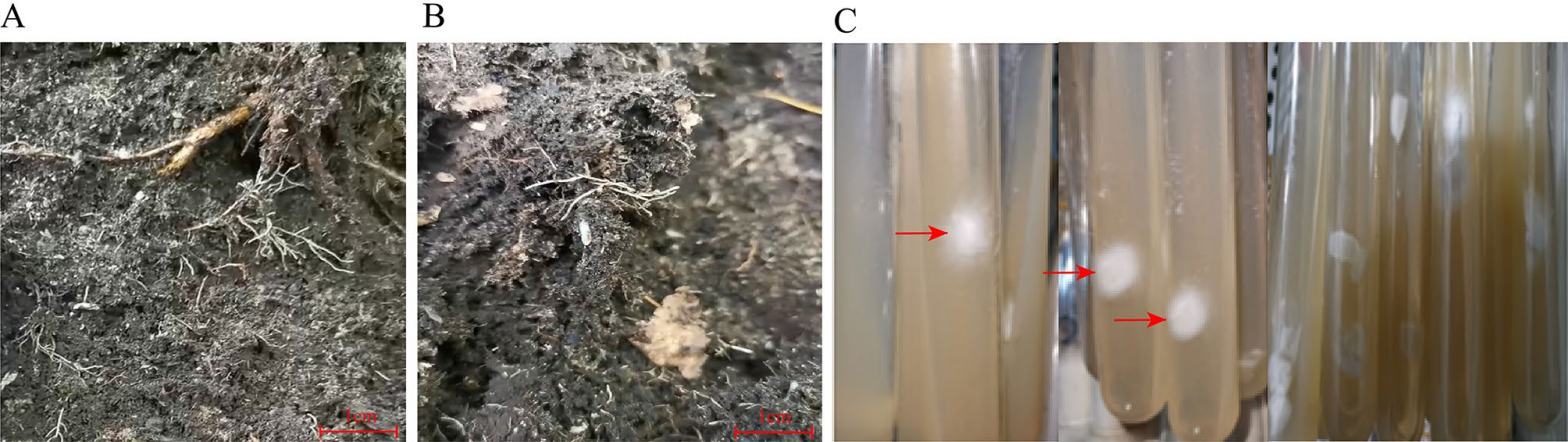
Eleven strains with single colonies were isolated and screened from the vegetative propagation corms. (**A** and **B**) Vegetative propagation corms grew in the sowing field; (**C**) eleven strains with single colonies had been isolated and screened from the vegetative propagation corms (the arrows indicated three strains that conformed to the morphological characteristics of germination fungus).

Eleven vegetative propagation corms that were symbiotic with the germination fungus were successfully obtained using *G. elata* Bl. *f. glauca* seeds (Fig. 3). Eleven strains with single colonies were isolated and screened from the vegetative propagation corms, and three strains were selected that conformed to the morphological characteristics of germination fungus (the germination fungus colonies were concentric circles, the mycelium was white and fluffy, the aerial roots were developed, and the middle was dense, with sparse edges and neat outlines), early germination (mycelium began to germinate 48–72 h after inoculation), and fast growth rate, and were named GYGL-1, GYGL-2, and GYGL-3 respectively.

### Results of the molecular identification of seed germination fungus from *G. elata* Bl. *f. glauca* seeds

**Figure 4:**
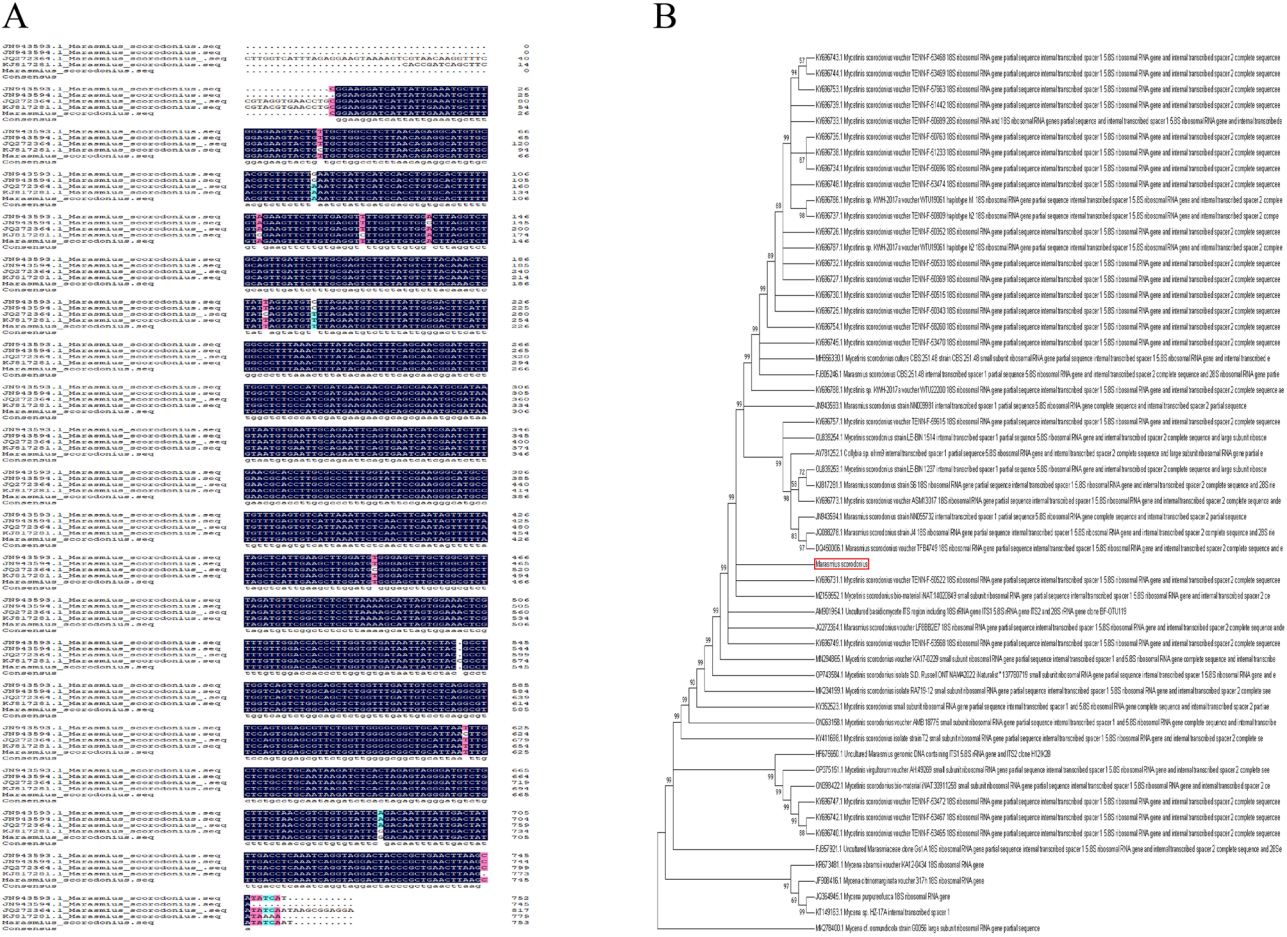
ITS sequence alignment and phylogenetic tree analysis. (**A**) The alignment results between the base sequence obtained from sequencing and the *My. scorodonius* sequence in NCBI (JN943593; JN943594; JQ272364; KJ817281); (**B**) The phylogenetic tree of the top 50 sequences with the highest homology.

Extracted the DNA of GYGL-1, GYGL-2, and GYGL-3, and used primers ITS1 and ITS4 to amplify the PCR products. After agarose gel electrophoresis, three clear and bright bands appeared at about 750 bp (Fig. S4), which was consistent with the length range of the fungal ITS sequence. The ITS sequences of GYGL-1, GYGL-2 and GYGL-3 were identical (Fig. S5). This showed that GYGL-1, GYGL-2 and GYGL-3 were the same strain. Therefore, GYGL-1 (OR399521) was selected for the next experiment. The homology of GYGL-1 to the reported *Mycetinis scorodonius* (JQ272364) was as high as 99.60% (Fig. 4A). The phylogenetic tree showed that GYGL-1 and *My. scorodonius* formed a stable phylogenetic branch (Fig. 4B). Therefore, GYGL-1 was initially identified as *My. scorodonius*.

### Results of the regenerated sporocarps identification of GYGL-1

**Figure 5:**
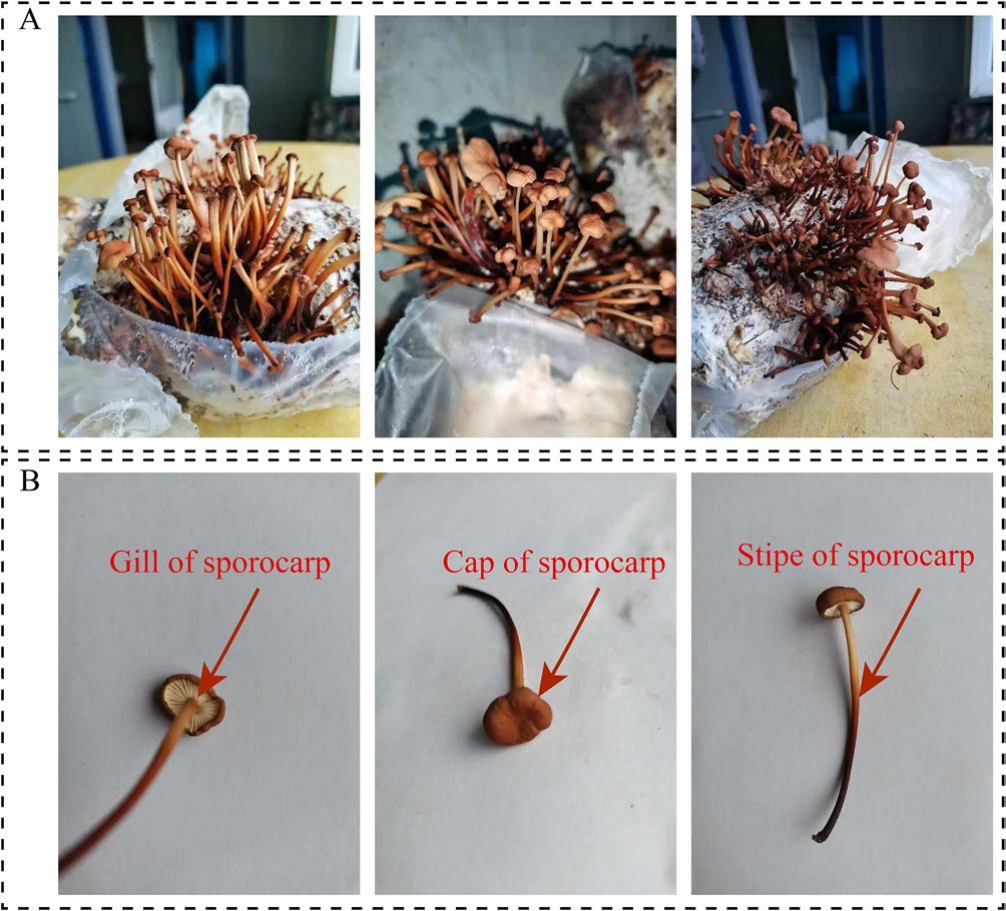
The sporocarps produced by GYGL-1. (**A**) *GYGL-1* regenerated sporocarps; (**B**) the gill, cap and stipe of the sporocarp.

After cultivation in a fungal bag, GYGL-1 regenerated sporocarps were successfully obtained (Fig. 5A). As depicted in Fig. 5B, the regenerated sporocarps of GYGL-1 were relatively small, with a cap diameter ranging from 0.5 to 3 cm. The cap had a flat hemispherical to spreading shape, initially with rolled–in margins that later stretched out. The surface appeared dry and could range from smooth to slightly undulating, displaying shades of yellowish–brown to brownish coloration. The fungus flesh was thin, the gills were straight to nearly free, the density varies from dense to sparse, and the lengths were also different. The stipe was 1.5–6 cm long, 0.05–0.8 cm thick, light yellow to brown, tapering at the lower part, cylindrical or flattened, dry, smooth, brittle to hard, consistent with the characteristics of the fruiting body of *Mycetinis scorodonius*.

The PCR amplified fragment of the DNA of the regenerated sporocarps of GYGL-1 was about 750 bp (OR418367) (Fig. S6), and its base sequence was the same as that of GYGL-1 (Fig. S7). It was further confirmed that the isolated GYGL-1 was *My. scorodonius*.

### Identification results of the germination function of GYGL-1

**Figure 6:**
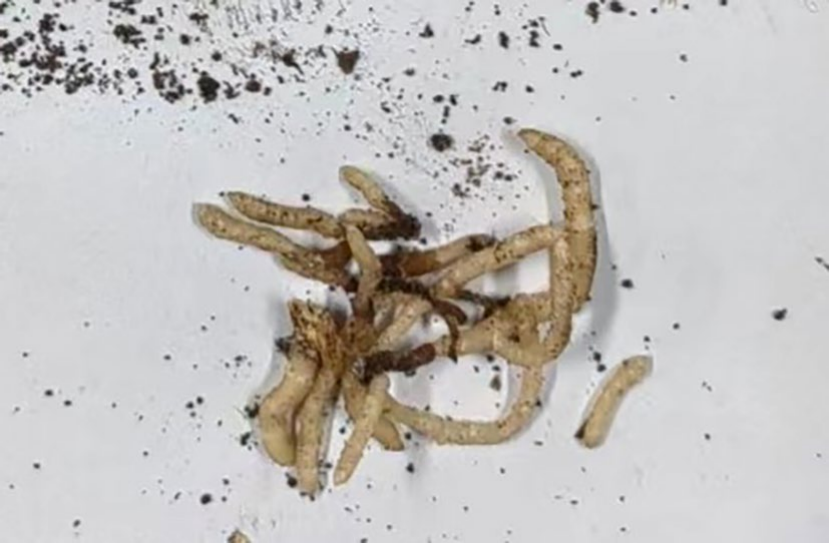
GYGL-1 and *G. elata* Bl. *f. glauca* seeds were co-cultured, successfully sprouting vegetative propagation corms.

After eight weeks of co-culture between GYGL-1 and *G. elata* Bl. *f. glauca* seeds, the vegetative propagation corms of *G. elata* Bl. *f. glauca* could be sprouted, proving that GYGL-1 had the function of promoting the germination of *G. elata* Bl. *f. glauca* seeds (Fig. 6).

### Comparison of growth rate between GYGL-1 and other germination fungus

**Figure 7:**
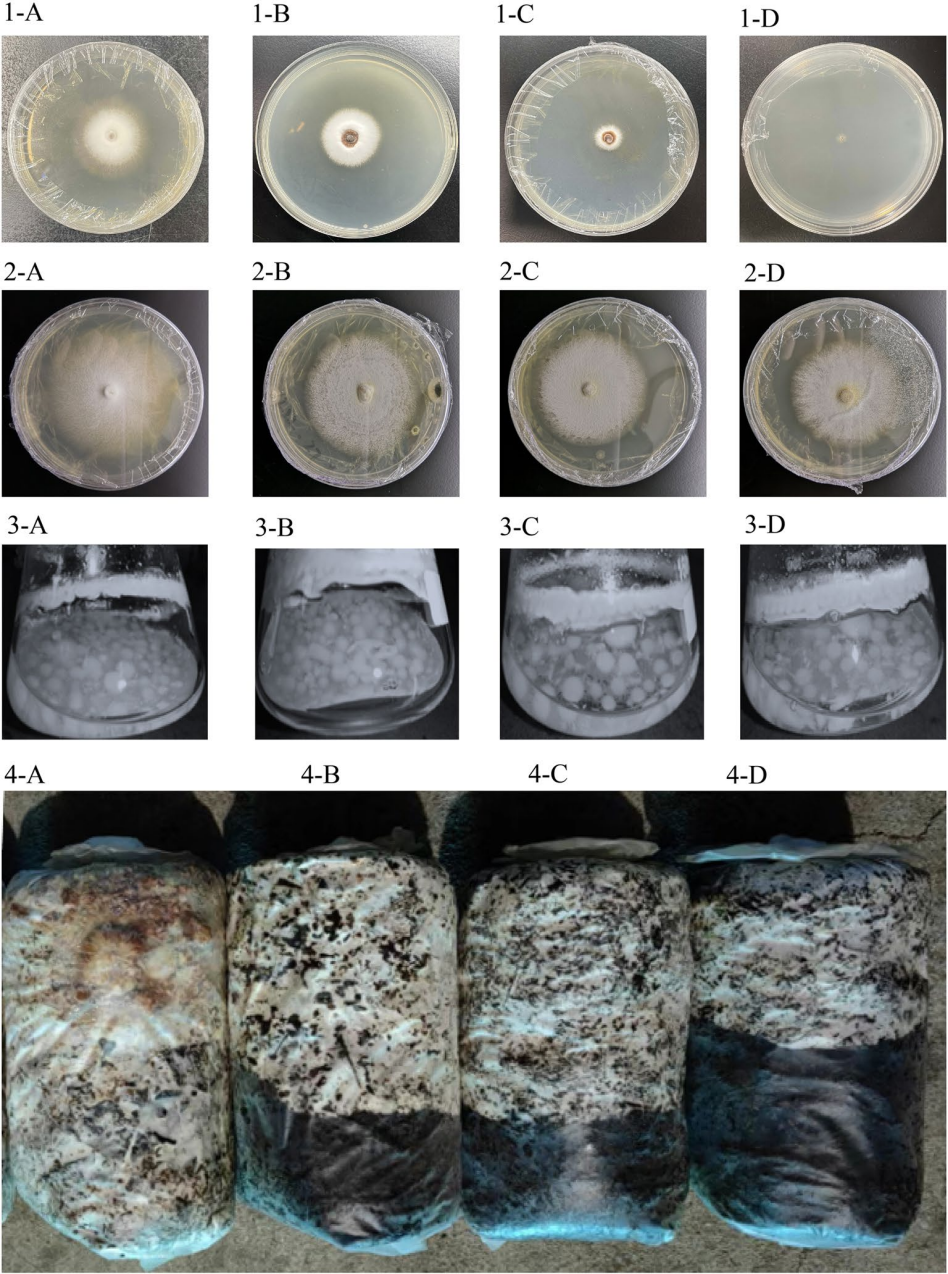
Comparison of GYGL-1 and other germination fungi. 1: Compared germination time after cryopreservation; 2: Compared mycelium growth rate on PDA medium; 3: Compared the weight of fungus ball in PDB medium; 4: Compared the time required for the fungus bag medium to be covered with the mycelium (**A**, GYGL-1; **B**, *M. purpureofusca*; **C**, *M. osmundicola*; **D**, *M. dendrobii*).

GYGL-1, compared with *M. purpureofusca*,* M. dendrobii*, and* M. osmundicola*, had the earliest germination time, only 2.5 days. The germination time was 32% shorter than *M. purpureofusca*, 58% shorter than *M. dendrobii*, and 53% shorter than *M. osmundicola*. (*P* < 0.05) (Table 1, Fig. 7-1). On PDA medium, the growth rate of GYGL-1 had increased by 16.48%, 90.05% and 49.77% respectively compared with the other three germination fungus (*P* < 0.05) (Table 1, Fig. 7-2). On PDB medium, the fungus ball weight of GYGL-1 had increased by 16.58%, 64.38% and 59.30% respectively compared with the other three germination fungus (*P* < 0.05) (Table 1, Fig. 7-3). In the cultivation seed bag medium, compared with the other three germination fungus, the time required for GYGL-1 to grow mycelium into the fungus bag was shortened by 25.67d, 40.17d and 32 days respectively (*P* < 0.05) (Table 1, Fig. 7-4). These results indicated that the growth rate of GYGL-1 was higher than that of the other three germination fungus.

**Table 1:** Comparison of GYGL-1 and other germination fungus (*P* < 0.05).

Germination fungus	GYGL-1	*M. purpureofusca*	*M. osmundicola*	*M. dendrobii*
Germination time (d)	2.50 ± 0.55^a^	3.67 ± 0.82^b^	5.33 ± 0.52^c^	6.00 ± 0.63^c^
Growth rate (mm/d)	3.25 ± 0.21^d^	2.79 ± 0.16^c^	2.17 ± 0.20^b^	1.71 ± 0.17^a^
Fungus ball weight (mg/bottle)	386.83 ± 30.56^c^	331.83 ± 27.10^b^	242.83 ± 28.74^a^	235.33 ± 29.06^a^
Time for mycelium to cover the fungus bag (d)	30.33 ± 3.67^a^	56.00 ± 3.69^b^	62.33 ± 4.50^c^	70.50 ± 6.28^d^

Data with the different letters in the same row are significantly different (*P* < 0.05).

### Comparative results of GYGL-1 and other germination fungus on seed germination ability and seedling growth of *G. elata* Bl. *f. glauca*

**Figure 8:**
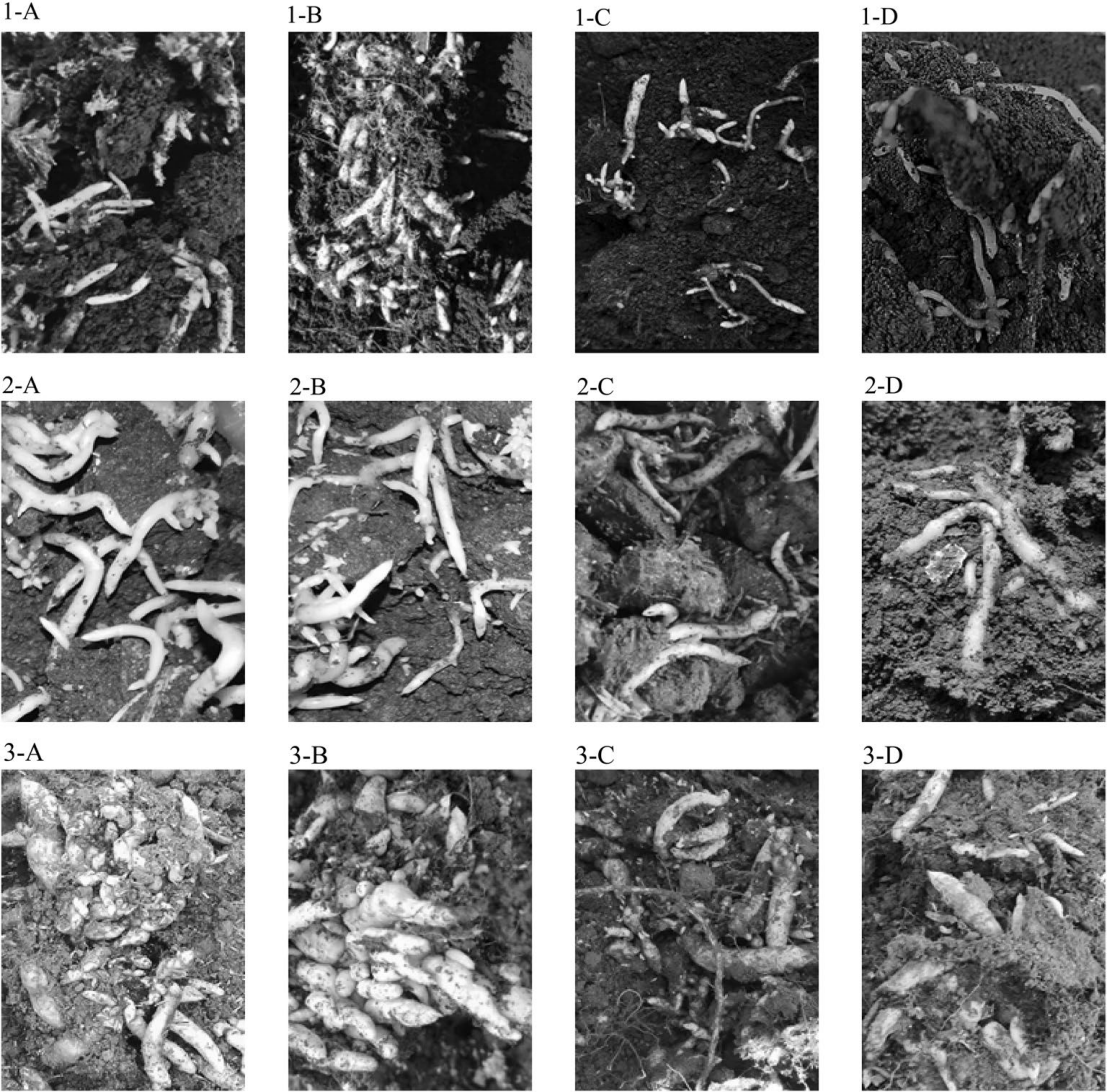
Comparison of GYGL-1 and other germination fungus on seed germination ability and seedling growth. 1: The effect of GYGL-1 and other germination fungus on the germination of indoor-cultured *G. elata* Bl. *f. glauca* seeds into vegetative propagation corms; 2: The effect of GYGL-1 and other germination fungus on the germination of field-grown *G. elata* Bl. *f. glauca* seeds into vegetative propagation corms; 3: The effect of GYGL-1 and other germination fungus on the germination of field-grown *G. elata* Bl. *f. glauca* seeds into immature tuber seedlings. (**A**) *M. purpureofusca*; (**B**) GYGL-1; (**C**) *M. dendrobii*; (**D**) *M. osmundicola*.

Comparing GYGL-1 with *M. purpureofusca*,* M. dendrobii*, and* M. osmundicola*, there were significant differences in the effects of GYGL-1 on the protocorms (germination rate) and vegetative propagation corms (number and weight) of indoor cultured *G. elata* Bl. *f. glauca* seeds. GYGL-1 was significantly higher than the other three germination fungus (*P* < 0.05) (Table 2, Fig. 8-1). There were significant differences in the effects of GYGL-1 on the protocorms (germination rate), vegetative propagation corms (number and weight) (Table 2, Fig. 8-2) and immature tuber seedlings (number and weight) cultured in the field of *G. elata* Bl. *f. glauca* seeds. GYGL-1 was also significantly higher than the other three germination fungus (*P* < 0.05) (Table 2, Fig. 8-3). It indicated that GYGL-1 was more suitable for the germination of *G. elata* Bl. *f. glauca* seeds in the Changbai Mountain region.

**Table 2:** Comparison of GYGL-1 and other germination fungus on seed germination ability and seedling growth.

Germination fungus	*M. purpureofusca*	GYGL-1	*M. dendrobii*	*M. osmundicola*
Indoor 5-week germination rate (%)	74.67 ± 5.82^c^	94.17 ± 4.12^d^	35.67 ± 3.20^a^	44.33 ± 3.33^b^
Indoor 8-week quantity (pieces/bottle)	21.00 ± 2.83^c^	32.00 ± 3.74^d^	6.00 ± 0.89^a^	15.17 ± 1.60^b^
Indoor 8-week total weight (mg/bottle)	236.67 ± 34.19^c^	403.67 ± 44.91^d^	57.67 ± 8.33^a^	173.50 ± 14.68^b^
Indoor 8-week single weight (mg/piece)	11.26 ± 0.36^b^	12.62 ± 0.08^c^	9.63 ± 0.70^a^	11.47 ± 0.50^b^
5-week germination rate in the field (%)	86.67 ± 2.11^c^	95.00 ± 2.24^d^	38.33 ± 3.07^a^	50.00 ± 2.58^b^
Number of 8-week in the field (pieces /60 g soil)	22.50 ± 1.54^c^	35.17 ± 2.29^d^	7.83 ± 0.70^a^	16.67 ± 0.88^b^
Total weight of 8-week in the field (g/60 g soil)	1.60 ± 0.18^ab^	2.67 ± 0.46^c^	0.55 ± 0.08^a^	1.12 ± 0.08^ab^
Single weight of 8-week in the field (mg/piece)	71.59 ± 7.44^a^	74.47 ± 10.01^a^	68.30 ± 5.02^a^	66.69 ± 1.40^a^
Number of 18 months in the field (pieces /m^2^)	637.83 ± 25.80^b^	714.67 ± 34.49^c^	561.00 ± 18.17^a^	550.83 ± 21.73^a^
Total weight 8 of 18-month in the field (kg/m^2^)	4.43 ± 0.20^b^	5.23 ± 0.21^c^	3.17 ± 0.20^a^	3.67 ± 0.25^a^
Single weight of 18 months in the field (g/piece)	7.02 ± 0.49^ab^	7.36 ± 0.27^b^	5.71 ± 0.45^a^	6.69 ± 0.48^ab^

Data with the different letters in the same row are significantly different (*P* < 0.05).

## Discussion

*G. elata* Bl. has an extremely high medicinal value; therefore, its demand shows an upward trend. Due to over-exploitation, the number of wild *G. elata* Bl. in China is declining, and the species is currently classified as “vulnerable” by the International Union for Conservation of Nature. Environmental conditions are the basis of plant reproduction and critical factors that control seed germination^27^. Environmental factors are the most important factors influencing the growth of *G. elata* Bl., including altitude and precipitation^28^. Therefore, different regions cultivate *G. elata* Bl. in different geographical locations and climates^28,29^, resulting in different varieties of germination fungus. In the cultivation of *G. elata* Bl., the commonly used germination fungus includes *M. osmundicola* and *M. dendrobii*, but they were all isolated and identified in central and southern China. These strains are unstable and severely degraded after multiple generations of reproduction in northeast China. It is particularly important to isolate new strains that promote the germination of *G. elata* Bl. seeds. The germination fungus isolated and identified in northeast China that can promote the germination of *G. elata* Bl. *f. glauca* seeds are more conducive to the production of *G. elata* Bl. *f. glauca*^25^. The Changbai Mountain region, where our experimental materials were collected, has significant regional characteristics because it is located at mid–to–high latitudes and is very sensitive to global climate change^30,31^. The affinity of the isolated germination fungus to *G. elata* Bl. *f. glauca* is more stable.

Over the past two decades, isolation methods for germination fungus have mainly consisted of finding sporocarps, wild protocorms isolation, and seed–capture protocorms, each with its advantages and disadvantages. Finding the sporocarp method, the technical steps are simple and quick^32^. However, the strains found in this way are accidental. Most strains do not have the function of germinating seeds and are inefficient. The results showed that few germinated seeds were found, which is not satisfactory for downstream production. Most fungi isolated by identifying wild protocorms can be used for seed germination. Compared with the the method of searching for sporocarps, the method of searching for wild protocorms is used to isolate germination fungus. Most of the isolated strains have the function of seed germination^33^. However, the protocorms are small and difficult to find, and the search process is very difficult. The method of capturing protocorms from seeds with well-defined targets has been widely used^34^. However, few strains isolated by this method can development of protocorms into vegetative propagation corms. This study uses a new method, which is to use seed-germinated vegetative propagation corms to capture germination fungus. This method is easy to find the vegetative propagation corms and has a high success rate in isolating germination fungus. Protocorms can develop into vegetative propagation corms, indicating that the germination fungus isolated by this method have higher germination ability for *G. elata* Bl.

Mycena (Pers.) Roussel, with approximately 600 species distributed worldwide, is one of the largest genera in Agaricales^35^. To date, fewer than 100 species of Mycena have been documented in China^36^,among them, only a few strains can be used for *G. elata* Bl. cultivation, including *M. osmundicola*, *M. dendrobii*, *M. purpureofusca*, etc. At present, our understanding of germination fungus is limited, and we have not yet identified any strains that can be used for the cultivation of *G. elata* Bl., with the exception of Mycena spp. In this study, we identified an effective germination fungus. Through morphology, molecular biology, sporocarps regeneration and functional identification, we identified the strain as *My. scorodonius*. This strain is reported for the first time to have the function of germinating *G. elata* Bl. *f. glauca* seeds. It is of great significance to the production of *G. elata* Bl.

In this study, *My. scorodonius* regenerated sporocarps were obtained using fungal bag culture. The germination fungus was identified through the regenerated sporocarps, which is not common among other isolated and identified strains. At the same time, due to the strong garlic odour of *My. scorodonius* sporocarps, it is useful for the development of *My. scorodonius* spices^37,38^. Compared with other current *G. elata* Bl. seed germination fungus, *My. scorodonius* requires the shortest germination time after cryopreservation, has a fast mycelium growth rate, high mycelium biomass, and high germination ability for *G. elata* Bl. *f. glauca* seeds, indicating that *My. scorodonius* can shorten the growth time of *G. elata* Bl. and increase the yield of *G. elata* Bl.

Some studies have shown the dynamics of the fungal community during the growth of *G. elata* Bl. It is believed that the growth of *G. elata* Bl. is also related to a variety of fungi other than Mycena^39^. These fungi change with the growth stages of *G. elata* Bl., such as Resinicium and Campanella^40,41^. However, these studies only analyzed the correlation between microbial species and *G. elata* Bl. germination, which are possible results of speculation, and there is still a lack of direct evidence. And there are many kinds of strains in the Mycetinis family. Just like the Mycena family, not all strains have the function of germination *G. elata* Bl. seeds. The specific strain has not been identified, and isolation and identification and germination function verification are still needed.

Although this study has done some meaningful work, there is still a lot of related and interesting research that needs to be carried out. For example, through multi-omics analysis, the interaction mechanism between *G. elata* Bl. seeds and germination fungus is analyzed, and how *G. elata* Bl. seed germination sends out signals for interaction with fungi. By comparing the germination fungus and non-germination fungus of the Mycena family, we can find out the substances that make *G. elata* Bl. seeds germinate from the germination fungus. To elucidate the degradation mechanism of multi-generation asexual reproduction of germination fungus and to study the regulation technology by comparing different generations of germination fungus in artificial culture. Through microbial isolation and identification and tieback experiments, we will verify whether the germination fungus is endophytic fungi of *G. elata* Bl. and whether they can colonize *G. elata* Bl. Isotope tracer techniques and molecular labelling techniques are used to investigate how germination fungus are spread in nature (spores, mycelium and *G. elata* Bl.). In this study, the combination of germination fungus and *A. gallica* promoted the growth of vegetative propagation corms of *G. elata* Bl. It inspired us to screen the different family of Mycena and Mycetinis that can be symbiotic strains by confrontation experiments, and to evaluate whether the effect of their combination on the germination of *G. elata* Bl. seeds can be enhanced by the germination seed experiments. The same method can also be used to study the effect of mixing germination fungus of the same family on the germination of *G. elata* Bl. seeds. All are good research directions.

### Supplementary Information

Supplementary Figures.

## Data Availability

The sequence data generated during the current study are available in the NCBI database under accession numbers OR399521 and OR418367.
