# Supplementary material for: Discovery of seed germinating fungi (Mycetinis scorodonius) from Gastrodia elata Bl. f. glauca S. chow in Changbai Mountain and examination of their germination ability
Source: Sci Rep. 2024 May 28;14:12215. doi: 10.1038/s41598-024-63189-3 (PMC11133366; doi:10.1038/s41598-024-63189-3)
Supplement: Supplementary file 1 — Supplementary Figures. [file 41598_2024_63189_MOESM1_ESM.docx]

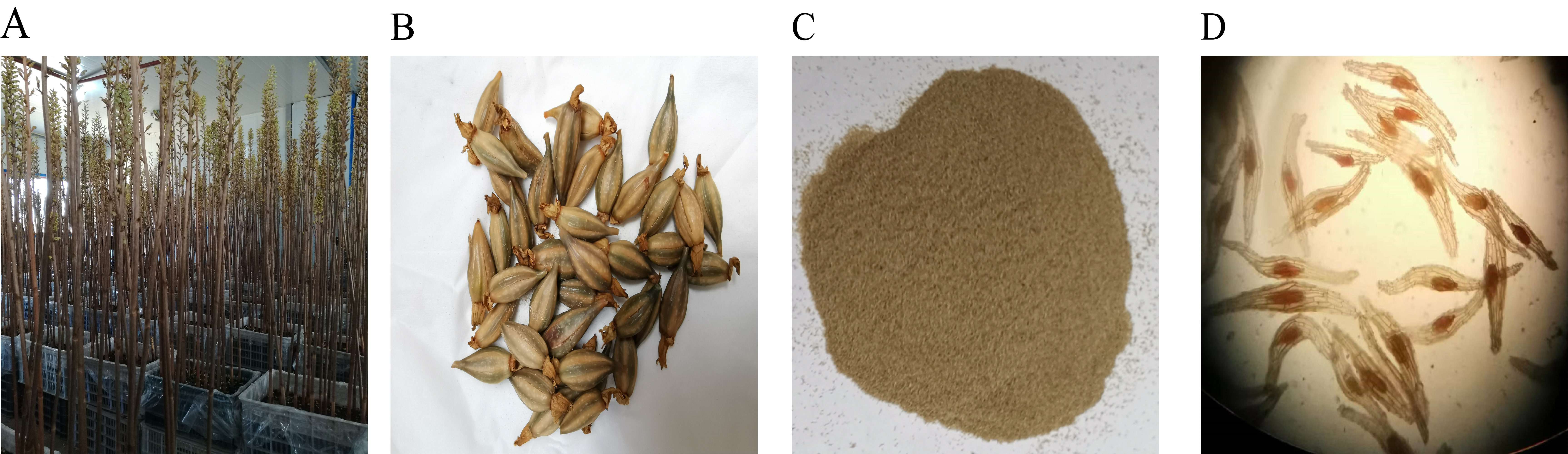


**Fig. S1.** **Seeds of *G. elata* Bl. used for seed-capture germination fungus.**

A, Stalk of *G. elata* Bl. *f. glauca*; B, Capsule of *G. elata* Bl. *f. glauca*; C, *G. elata* Bl. *f. glauca* Seeds; D, Viability determination of *G. elata* Bl. *f. glauca* seeds.


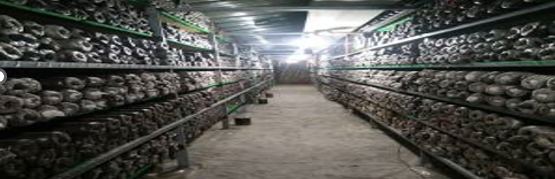


**Fig. S2. The large-scale culture of germination fungus and the culture of regenerated sporocarps.**


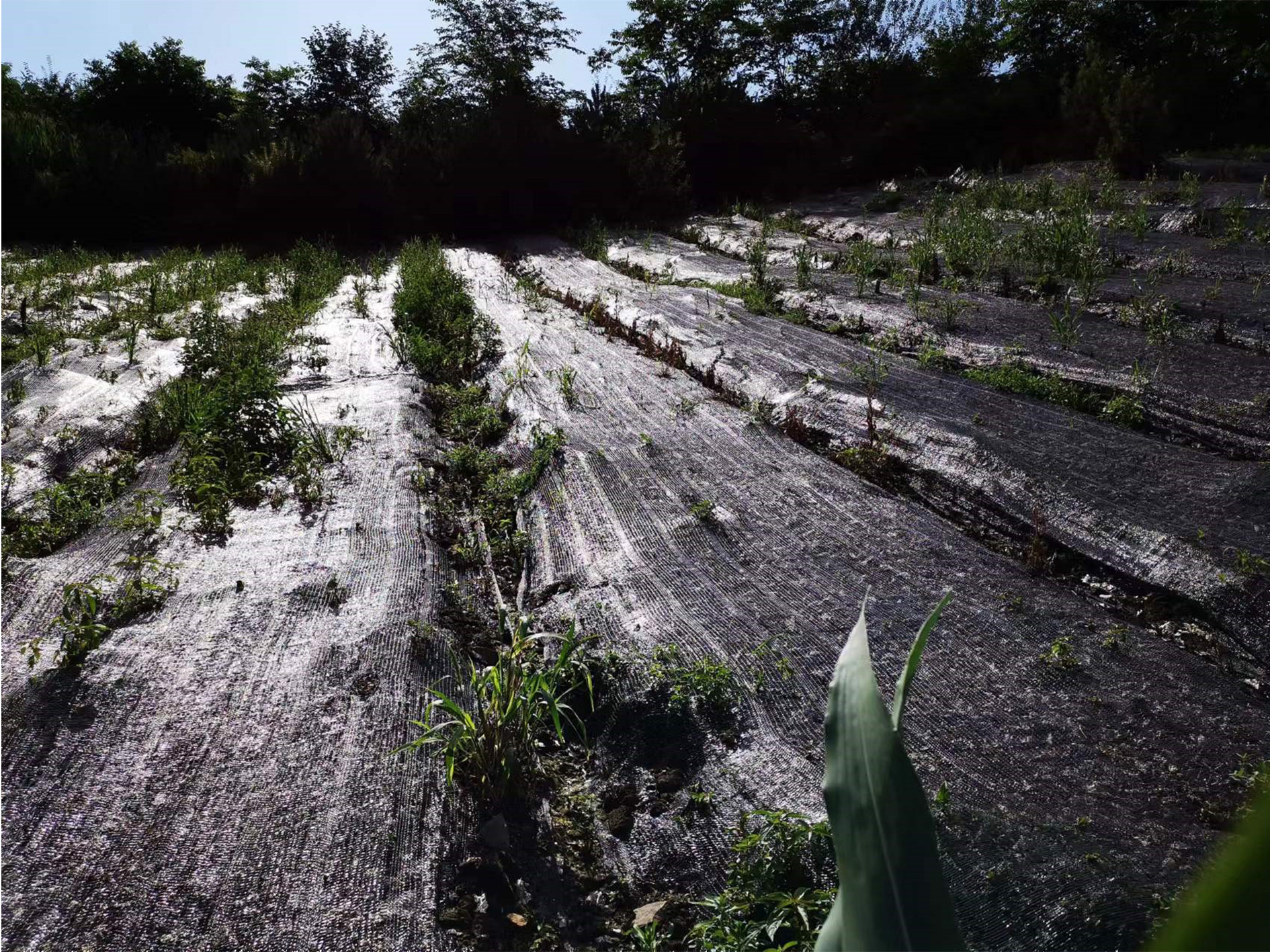


**Fig. S3. Field photographs of the production verification experiment on seed germination of *G. elata* Bl. *f. glauca*.**


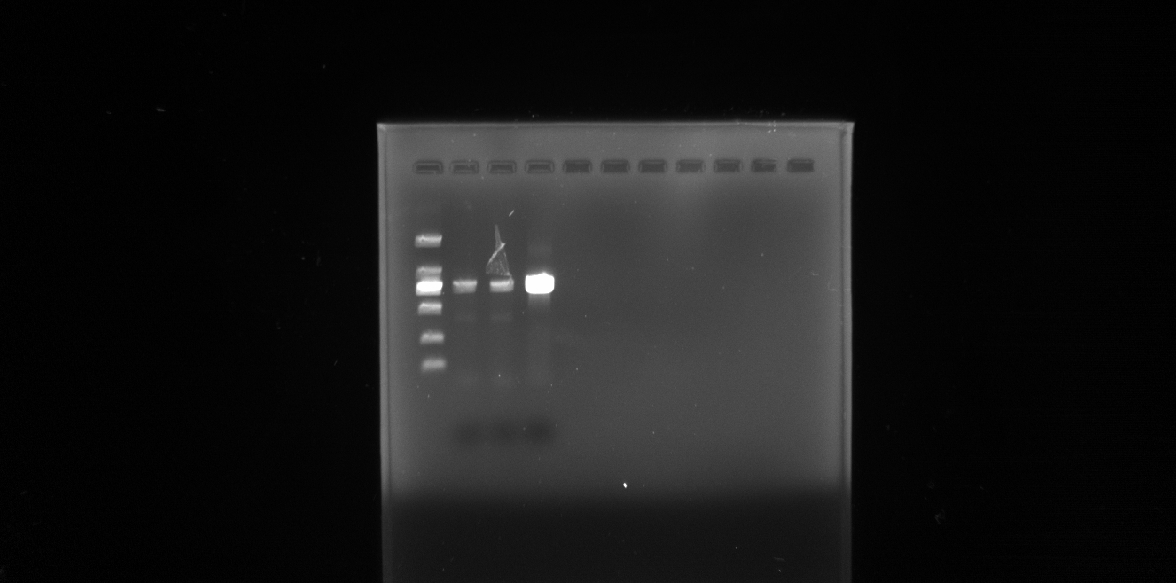


**Fig. S4. Agarose gel electrophoresis pattern of GYGL-1, GYGL-2, and GYGL-3.**

**Fig. S5. ITS sequence alignment of GYGL-1, GYGL-2, and GYGL-3.**


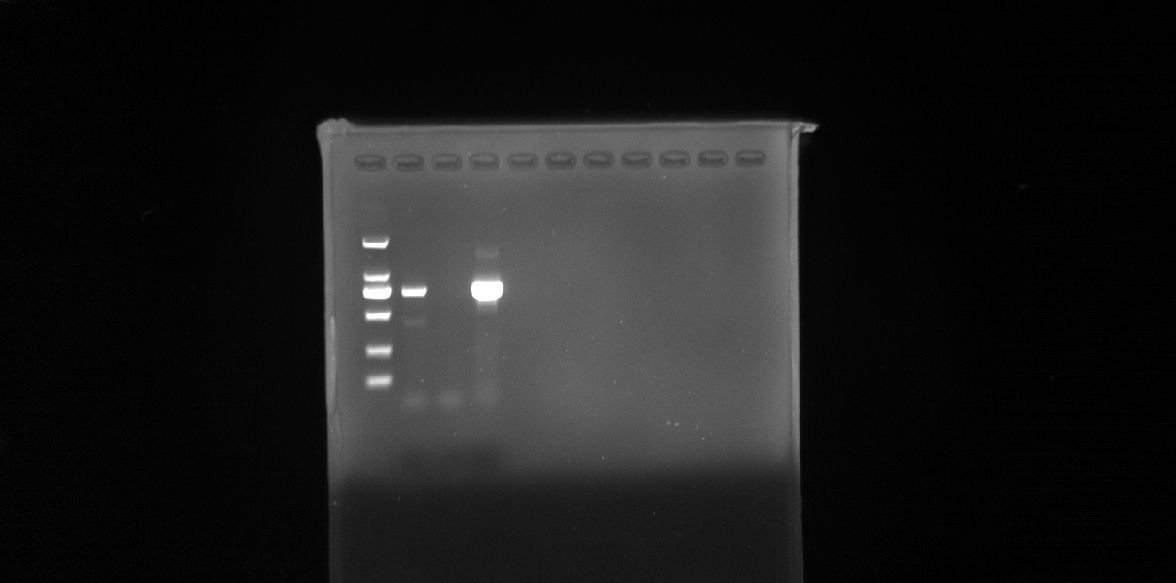


**Fig. S6. The ITS sequence of the regenerated sporocarps.**

**Fig. S7. ITS sequence alignment of OR399521 and OR418367.**
